# Virome in the Lungs: The Role of Anelloviruses in Childhood Respiratory Diseases

**DOI:** 10.3390/microorganisms9071357

**Published:** 2021-06-23

**Authors:** Giulia Dodi, Marina Attanasi, Paola Di Filippo, Sabrina Di Pillo, Francesco Chiarelli

**Affiliations:** Pediatric Allergy and Pulmonology Unit, Department of Pediatrics, University of Chieti-Pescara, 66100 Chieti, Italy; giulia.dodi16@gmail.com (G.D.); difilippopaola@libero.it (P.D.F.); sabrina.dipillo@gmail.com (S.D.P.); chiarelli@unich.it (F.C.)

**Keywords:** lung microbiome, lung virome, chronic respiratory diseases, children, asthma, cystic fibrosis, bronchiectasis, anelloviruses, torque teno virus

## Abstract

More recently, increasing attention has been directed to exploring the function of the global virome in health and disease. Currently, by new molecular techniques, such as metagenomic DNA sequencing, the virome has been better unveiled. By investigating the human lung virome, we could provide novel insights into respiratory diseases. The virome, as a part of the microbiome, is characterized by a constant change in composition related to the type of diet, environment, and our genetic code, and other incalculable factors. The virome plays a substantial role in modulating human immune defenses and contributing to the inflammatory processes. Anelloviruses (AVs) are new components of the virome. AVs are already present during early life and reproduce without apparently causing harm to the host. The role of AVs is still unknown, but several reports have shown that AVs could activate the inflammasomes, intracellular multiprotein oligomers of the innate immune system, which show a crucial role in the host defense to several pathogens. In this narrative revision, we summarize the epidemiological data related to the possible link between microbial alterations and chronic respiratory diseases in children. Briefly, we also describe the characteristics of the most frequent viral family present in the lung virome, Anelloviridae. Furthermore, we discuss how AVs could modulate the immune system in children, affecting the development of chronic respiratory diseases, particularly asthma, the most common chronic inflammatory disease in childhood.

## 1. Introduction

Nowadays, there is increasing interest in the characterization of microorganisms in human pathology. While the bacterial flora and its importance in human pathology are widely discussed in the literature, less is known about the human virome. This represents a new unexplored topic. The human virome is a changeable part of a microbial ecosystem. It is composed of eukaryotic viruses, bacterial viruses, the so-called “bacteriophages”, and a group of virus-derived genetic elements integrated into host chromosomes (human endogenous retroviruses, endogenous viral elements, so-called “prophages”) [1]. Eukaryotic viruses infect eukaryotic cells, and bacteriophages target specific human-hosted bacteria [1]. While both bacterial and viral components found in the gastrointestinal tract are considered not only pathogens but sometimes commensals, the respiratory tract was thought to be sterile. In fact, for a long time, any type of microbial finding from airway samples was thought to be an acute or exacerbated infective disease or interpreted as probable test contamination coming from the nasopharynx and oral cavity. Thanks to improvements in microbiological techniques, new local viral communities were discovered in the airways [2]. Recently, several studies showed that a regular lung microbiome is present, regardless of any inflammatory or infectious setting [3,4,5]. Contrary to other environmental viromes, the airways virome presents a relatively small range of species; one of the reasons for the low viral diversity is the presence of natural barriers. The natural barriers, including physical ones such as ciliary movements, and both innate and adaptive immunity, may end in low viral diversity. At the same time, viral pathogens (e.g., influenza, parainfluenza, rhinovirus (RV), respiratory syncytial virus (RSV), or adenovirus) can transitorily enrich the respiratory virome [4]. Another characteristic of a healthy respiratory microbiome is large inter-individual differences. In addition, in the case of specific diseases, microbial changes could represent a diagnostic sign, defining an acute situation or the flare-up of a chronic one [5]. Respiratory virome elements can be organized into two major groups: commensals and opportunistic pathogens. As it happens for bacterial infections, being a commensal virus or becoming a pathogen virus depends on many factors of the host and the virus itself. For example, local growth factors and environmental features of the airway can affect and modify the composition of the respiratory virome [1,4,6]. According to the literature, in asymptomatic individuals, transitory viruses (e.g., RSV or adenovirus) could provoke a low-level immune response, while increased loads of viruses and a higher level of inflammation were proven in patients with chronic airway disease [4]. Moreover, it seems that viral species of the respiratory tract are important in order to control other pathogens, particularly bacteriophages. Specifically, human-hosted airway bacteriophages could interfere with health and disease states and interact with the immunity system, by controlling their bacterial host. Various studies showed that bacteriophages provide a reservoir of virulence genes [7,8]: in fact, bacteriophages encode several bacterial virulence factors, such those that determine bacterial strength and the ability to colonize, adhere, invade, and produce toxins [9]. In addition, bacteriophages allow the conservation of antimicrobial resistance genes [7,8,9,10,11,12].

In other words, the human virome represents a part of the whole human microbiome and consists of pathogenic viruses, non-pathogenetic viruses that are found in healthy individuals, bacteriophages, and retroviral elements that are integrated into the human chromosomes. It is likely that the delay in understanding the virome depends on the absence of specific instruments to characterize viruses promptly and precisely, in contrast to the rapid technology techniques discovered to identify bacteria over recent decades [9,13]. Isolating viruses and bacteriophages still requires difficult and numerous preprocessing actions, even if we have new tools [14].

However, thanks to new technologies, previously unknown viral agents, such as anelloviruses, have been discovered as part of the lung virome of healthy individuals, in chronic respiratory diseases, and in circulating blood [15,16].

In this review, we will focus on how anelloviruses could modulate the host immune systems influencing the development of chronic respiratory diseases in childhood.

## 2. Search Strategy and Inclusion Criteria

References for this review were identified through searches of PubMed and Google Scholar for articles published from January 2001 to May 2021 using terms such as “lung microbiome”, “lung microbiota”, “lung virome”, “chronic respiratory diseases”, “children”, “asthma”, “Cystic Fibrosis”, “bronchiectasis”, “Anelloviruses”, and “Torque teno virus”. Older studies were only considered to report the first discoveries about anelloviruses. Articles were identified as interesting when the studies provided new insights regarding the role of the lung microbiota in childhood respiratory diseases. Articles resulting from these searches and the relevant references cited in those articles were reviewed. We only included articles published in English.

## 3. Anelloviruses

The family Anelloviridae includes 14 genera and is widespread in mammals and birds [17]. Alphatorquevirus, with torque teno virus (TTV), resulted in being the most frequent genus associated with the development of respiratory diseases [17].

In 1997, TTV was isolated for the first time from a Japanese patient with post-transfusion hepatitis of unidentified etiology [18]. Successively, the molecular and biophysical characterization of TTV identified a non-enveloped, single-stranded DNA virus with a circular configuration and a genome with a negative polarity [19]. TTV is the archetype of anelloviruses, and a large spectrum of genetically similar viruses exist, such as torquetenominivirus and torquetenomidivirus.

Initially postulated as a cause of cryptogenic hepatitis, the association between TTV infection and human hepatitis became questionable when a similar prevalence of TTV infection between Italian blood donors with a normal alanine aminotransferase level compared to those with an increased one was found [20]. The following studies confirmed that TTV infection is very common worldwide even in subjects without liver disease [21,22].

**Figure 1:**
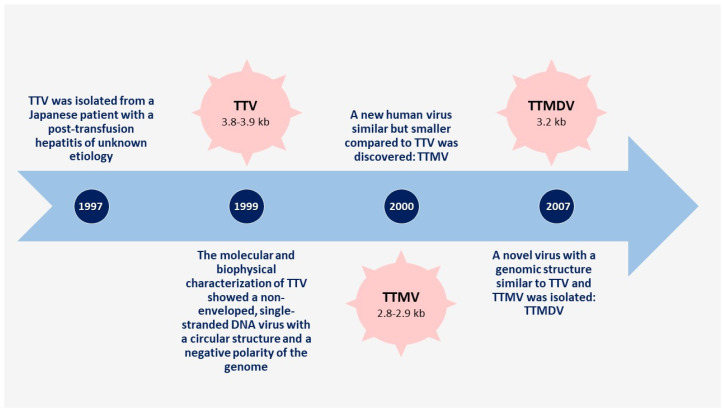
Timeline of human anelloviruses’ history stages since their discovery in 1997.

In 2000, a new human virus similar to TTV was discovered; it was named torque teno-like mini virus (TTMV) for its smaller size compared to TTV [21]. In 2007, a new virus with a genomic structure similar to TTV and TTMV was isolated and was named torque teno-like midi virus (TTMDV) [23]. The timeline of the discovery of AVs is shown in Figure 1.

These three anelloviruses can infect humans and were classified into the Alphatorquevirus (TTV), Betatorquevirus (TTMV), and Gammatorquevirus (TTMDV) genera of the Anelloviridae family [24]. The major differences between them are the genome size, which ranges from 3.8 to 3.9 kb for TTV, 3.2 kb for TTMDV and 2.8 to 2.9 kb for TTMV, and the nucleotide sequence, which is very different both among themselves and compared with AV types [25].

AVs and AV antibodies are widespread in humans at any age and, ubiquitously, suppose a commensality state [26]. Their spread occurs mainly through fecal–oral transmission, but mother–child and respiratory tract transmissions were also reported in the literature [25,27]. Infection with one or more of the three human AVs was demonstrated in 75–100% of tested subjects [28], and the infection could involve children from the first months of life [29].

Serum TTV positivity in the general population varies enormously between countries and it is estimated to be over 50%. The highest infection prevalence is found in patients undergoing transfusions, or hemodialysis, hemophiliacs treated with a clotting factor, or intravenous drug users. TTMV and TTMDV prevalence is less known, but it ranges from 40% to 80% of the general population [30].

Anellovirus replication can occur in several tissues/organs. Indeed, the presence of these viruses was demonstrated in blood, saliva, breast milk, stool, nasopharyngeal aspirates, and organs such as the thyroid gland, liver, spleen, pancreas, and many others [25].

Anelloviridae usually replicate at a low but stable state, not causing human disease [26]. The human immune system controls their replication [30]. In fact, they are inversely associated with levels of immunocompetence, reaching high blood levels in the case of immunosuppression [31] as in HIV patients [32] and in lung transplant recipients [33,34].

Therefore, it was recently hypothesized that they could be used as an immunosuppression marker [35] and as a predictor of clinical results in pediatric lung transplantation stratifying recipient risk [26].

Recently, AVs have been studied in various diseases. For instance, AVs seem to be responsible for a portion of fever of unknown origin in children [25,36]. Noteworthy, associations of AVs with chronic respiratory diseases, such as asthma or bronchiectasis, are also reported in the literature [37]. Unfortunately, their ubiquitous nature makes it difficult to define whether this association is really causal [25,36].

## 4. Anelloviruses and Immunity

The relationship between AV infection and human immunity is still to be clarified. Several studies found an association between AV load and immune response [26,32,33,34,35]. For example, low AV genome copies seem to be associated with transplant rejection or death in children who were transplanted [26], and it was suggested that human AV DNA load increases with the dosage of immunosuppressive therapy [34]. In a multicenter study performed in 2018, blood samples from 57 children included in the Clinical Trials in Organ Transplantation in Children (CTOT-C03) cohort were collected: patients with low alphatorquevirus levels at two weeks post-transplantation presented a higher probability to progress to acute rejection within three months after transplant, and low betatorquevirus levels at six weeks and six months after transplant were related to death and the composite outcome, represented by death, chronic rejection, or retransplant within 2 years [26]. Another immunosuppression state which was associated with AVs is HIV infection. HIV-1-positive patients presented high TTV and TTMV loads, with higher levels in subjects with AIDS [38]. Thom and Petrick [38] investigated the TTV and TTMV viral loads in the bone marrow and spleen of HIV-negative individuals, HIV-positive individuals, and HIV-positive individuals who had developed AIDS: the titers of the AIDS-affected group were meaningly higher compared with both the HIV-positive and negative groups both in the bone marrow and the spleen [38]. Moreover, Shibayama et al. [32] showed an inverse association between TTV levels and CD4 + T lymphocyte levels in HIV-1-positive persons: they investigated the prevalence and relative titer of TTV DNA among 144 patients with HIV infection by polymerase chain reaction (PCR) methods and found a higher prevalence of TTV DNA in HIV-infected patients than in controls (91% versus 27%); they also found a higher titer in HIV patients infected with AIDS, those with a low CD4 T cell count, or those with high HIV viral loads [32]. On the other hand, other studies [39,40] did not confirm these data. In their study, Nasser et al. [39] used primers for the detection of 197 bp localized in a region of the largest open reading frame (ORF-1) of the virus, in order to determine TTV DNA prevalence in HIV-infected patients; they found a prevalence of TTV DNA in HIV-infected patients of 12.5% and 6% among controls. The link between TTV prevalence and HIV-1 plasma viral load was also analyzed: there was no evidence of the association of TTV with CD4+ and CD8+ T cells in both asymptomatic subjects with a low HIV load and symptomatic patients with a high HIV load. Moen et al. [40] also found that CD4 + counts were not significantly consistent with the variations in TTV and TTMV concentrations.

The mechanism by which the virus interacts with our immune system is unknown.

Airway epithelial cells (AEC) are the first line of defense of the innate immune response, acting as sentinel cells in response to infection and constituting a physicochemical barrier. Pathogen-associated molecular patterns (PAMP) on the surface of infectious agents are recognized through pattern recognition receptors (PRR), e.g., Toll-like receptors (TLRs) and intracellular RNA helicases, and activate innate immune responses [41,42,43,44,45,46,47]. Other cells such as macrophages (AM) and dendritic cells (DC) are also involved [48]. The interaction among viruses and dendritic cells is mediated by several PRRs including TLRs (and TLR-9 is the most important); it seems that this microorganism–host relation has an effect on the balance among Th1, Th2, or Th17 subtypes, particularly in the respiratory airways [41,42,43,44,45,46]. One of the mechanisms proposed is that microbial products connected to TLRs on airway epithelial cells facilitate the discharge of the IL-7-like cytokines and thymic stromal lymphopoietin, while microbial components, interacting with TLRs on DCs, increase the expression of costimulatory molecules. The final result is a shift towards Th2 inflammation [47,48]. Another actor in the interaction between viruses and the immune system is micro-RNAs (miRNAs), both cellular and viral. Cellular miRNAs can block the translation of the viral genes, resist the block of apoptosis, and then contrast the persistent replication; in animal models, they can also polarize macrophages toward an allergic pattern of inflammation [49,50]. Viral miRNAs modulate the expression of viral genes and therefore also the interaction with the immune system and the resulting inflammation. However, miRNAs probably have a very complex role in inflammation: in fact, they are reported as both up-regulators and down-regulators of inflammation in several diseases, including asthma [50].

In regard to TTVs, it seems that TTVs come in contact with many pathogen-associated molecular pattern (PAMP) receptors (PRR), activating immune and inflammatory responses [51,52,53,54]. TLRs in infected cells are also stimulated by the genome of TTVs and its replication intermediates (unmethylated heterodimers of guanosine and cytosine (CpGs)); indeed, it has been reported that when TLR-9 detects CpGs, it triggers the release of inflammatory cytokines, such as IFN-α, interleukin (IL)-6, and IL-12. At the same time, TLR-9 can also generate an inhibitory signal [51,54,55]. Since both stimulatory and inhibitory CpGs are present in the DNA of TTV (as well as in most pathogens): the amount of stimulatory and inhibitory CpGs affects the interaction with TLR9 and therefore the development of inflammation [54,55]. In particular, TTV genogroup 4 is rich in stimulatory CpGs and activates TLR-9 in mouse spleen cells in vitro, enhancing the release of pro-inflammatory cytokines [51]. Maggi et al. [27] analyzed the presence, type, and load of TTV in nasal and plasma specimens by PCR assay in blood and nasal samples of more than one hundred children with ARD (acute respiratory distress): they found that the average loads of TTV were significantly higher in patients with pneumonia rather than in patients with less severe disease; specifically, TTV group 4 was detected only in the case of pneumonia [27]. Furthermore, TTV can shape the immune system by the interaction with inflammasomes and intracellular multiprotein complexes, which promote the tolerance or the susceptibility to infectious agents [52].

Concerning miRNAs, Kincaid et al. [53] showed that TTVs encode miRNAs which act on transcripts of their host cell, and that one of these miRNAs down-regulates the transcription of the NMI protein, which is associated with the interferon pathway and cytokine production in response to viral infection [53]; therefore, the authors suggested that TTV miRNAs allow viral persistence and immune escape.

In addition, the type of miRNA produced is different in ill patients [55,56]. In particular, the presence of TTV DNA and miRNA expression was evaluated in plasma samples of 77 diseased patients (20 with HIV infection, 18 with hepatitis B virus infection, 18 with hepatitis C (HCV) virus infection, and 21 solid organ transplants), and 25 healthy subjects: TTV prevalence was significantly higher in sickly patients (80%, 62/77) than in controls (60%, 15/25), and genetic TTV analysis showed a different prevalence of different miRNAs [55]. Noteworthy, miRNA production and release did not correlate with virus replication [55]. However, the role and the significance of TTV miRNAs, as well as the general role of miRNAs in the inflammatory pattern, are not yet completely understood.

Once in the bloodstream, TTV virions interact with immunoglobulins to form antibody–virus complexes, triggering the humoral immune response [57,58,59,60,61]. The principal target for immune recognition is the open reading frame (ORF) 1 protein, the putative protein forming the capsid of the virus. It seems that the ORF1 protein and the ORF2 protein, which is thought to be a phosphatase, are recognized by antibodies. Antibodies against ORF1 and ORF2 were found in people with a detectable TTV load [58]. On the other hand, it was demonstrated that AVs have developed some specific mechanisms to persist in the host: ORF2 can suppress Nuclear Factor κB (NFκb) translocation to the cell nucleus and, consequently, its capability to promote transcription of genes, such as IL-6, IL-8, and cyclooxygenase-2, reducing the host inflammatory response [59]. More recently, it was found that TTV particles can spread in the body within the exosomes, increasing the capacity of diffusion of the virus: exosomes allow the entry of TTV in otherwise non-permissive cells, and the virus within them may be protected from neutralizing antibodies [62]. A summary of the aforementioned mechanisms is provided in Table 1 and Table 2.

## 5. Anelloviruses and Chronic Respiratory Diseases

The global incidence of chronic lung diseases (CLDs), as well as the socio-economic burden derived from them, is rising [63,64]. Respiratory microbiomes (bacterial microbiome, virome, mycobiome, parasitome) during CLDs would result in being impaired and easily affected by several environmental factors (air pollution, allergens, infections, and cigarette smoke) [65]. However, further research is needed to better investigate which risk factors impair the diversity of the microbiome in the lungs. Furthermore, it is generally thought that in early life, factors influencing the airway microbiome, such as diet, the environment, and genetic background, might induce the development of the pulmonary immune system. A microbial imbalance during growth may lay the groundwork for subsequent lung disease [66].

Among CLDs, asthma is the most frequent chronic inflammatory disease in childhood, and its annual incidence is increasing [64,67,68,69]. Phase III of the International Study of Asthma and Allergy in Childhood (ISAAC study) showed that the worldwide prevalence of current wheezing increased, on average, by 0.06% per year in the 13–14 year age group, and by 0.13% per year in the 6–7 year age group [67]. Despite the increasing burden of this lung pathological condition, currently, the therapeutic options are scarce. Inhaled corticosteroids (ICs) remain the cornerstone for the therapy of children with persistent asthma, although a small proportion of asthmatics are resistant to ICs [70]. A natural history of asthma is characterized by exacerbations which are one of the most common causes of hospital admissions in children [68,69]. Although the role of viruses as triggers of asthma flare-ups has been widely studied [71,72,73,74,75], recent studies have investigated a potential link between some new viral families, such as AV and Redondoviridae, and chronic respiratory diseases in children, particularly in asthma [27,37,76].

However, mechanisms causing asthma exacerbations are still not fully understood and are different among children [77,78]. Hence, a better comprehension of the underlying pathogenic processes could lead to further treatment options [78,79]. The introduction of non-culture-based methods (i.e., next-generation sequencing technologies) has yielded the detection of previously unknown human pathogens [80,81], leading to better investigating the host–microbiome interactions and specifically the part of the virome in lung diseases [80]. Potential drugs might be obtained for manipulating the lung microbiome, using, as targets, specific microbes or their products (toxins, metabolites) [82]. Zhou et al. [83] demonstrated that 33% of samples collected in asymptomatic children with asthma contained viruses, and most of them were rhinoviruses (64%). In addition, the presence of viruses in the samples at the baseline was not predictive of the following asthma exacerbations [83].

Similarly, in 211 nasopharyngeal samples obtained from children with asthma flare-ups, viruses were identified in 20% of the study population, specifically rhinovirus, RSV, and enterovirus [84].

Using a metagenomics approach, a viral dysbiosis was also evident in nasopharyngeal aspirates of asthmatic children, characterized by a decrement of bacteriophages and an increase in viral families, mostly represented by AVs and picornaviruses [85,86].

Nowadays, it is known that the patients susceptible to asthma exacerbations present a definite phenotype characterized by, or an airway inflammation related to, an asthmatic process, such as an inflammatory pattern derived from the response to pathogens [77]. However, whether the airway inflammation related to an asthmatic process primes the pathogenic effect of the viral infection, or whether the virus yields an exaggerated inflammatory response is still much discussed, and this question has been a pivotal matter of several studies over the past 25 years [45,71,72,73,74,75]. Actually, no specific harmful effect has been attributed to any AV thus far [87]. However, the presence and the high load of TTV have been associated with some acute respiratory diseases both in children and in adults [27,88]. In adults, Xie et al. [89] showed a high prevalence of distribution of torque teno virus (95.6%) in the lungs of 91 patients with COPD, revealing that the observation rate of TTV in COPD is higher than that of other chronic respiratory diseases. Interestingly, the authors stated that an alteration of the immune system in COPD patients could contribute to the high prevalence of TTV, reflecting the immunocompetence of hosts [88,89].

More recently, in adult patients with coronavirus disease 2019 (COVID-19), the respiratory microbiome was investigated to detect potential changes in the bacterial communities and commensal viruses, such as AVs and redondoviruses, in association with this pathological condition [89,90]; specifically, the authors collected 507 oropharyngeal, nasopharyngeal, and endotracheal samples from 83 hospitalized COVID-19 patients and 75 samples from 13 critically ill patients hospitalized for other diseases, showing an interesting dysbiosis of the lung microbiome in hospitalized patients with COVID-19 compared to other patients. Noteworthy, the authors found that AVs and redondoviruses resulted in being important factors to distinguish patients that were intubated or not [89,90].

In the pediatric population, children with pneumonia showed visibly higher TTV loads than children with a milder lung infection [27,90]. Maggi and Bendinelli [91] suggested that TTV, probably in combination with other viruses, might act as an “strengthener” of the inflammatory mechanism or at the systemic level at specific body sites (upper and lower respiratory tracts). The authors stated that the airways could be a primary site of infection and replication in infants with acute respiratory disease [92]. Pifferi et al. [90] found an important association between lung function parameters and nasal TTV load in children with asthma and no association between them and nasal TTV load in healthy controls. Specifically, the authors showed abnormal forced expiratory flow between 25 and 75% (FEF25–75%) of forced vital capacity (FVC) values more frequently in asthmatic children with a high nasal TTV load compared to those asthmatic children with a low TTV viral load. In addition, other spirometric indices, such as the forced expiratory volume in 1 s (FEV1)/FVC ratio and FEF25–75%/FVC ratio, were significantly lower in asthmatic children with a high TTV load than those asthmatic children with a low TTV load [90]. The link between TTV load and airflow obstruction was confirmed in a group of patients affected by radiologically detected bronchiectasis: there was an association of TTV loads with FEF25–75% and the FEF25–75%/FVC ratio. In patients with bronchiectasis, a higher prevalence of TTV was found compared to controls (96% versus 45%) [92]. In addition to the association with impaired pulmonary function, high TTV loads might contribute to lymphocyte imbalances during respiratory disease [93]. In the peripheral blood of 40 children with ARD, there was an inverse correlation between the percentages of total T lymphocytes (CD3) and T helper, and the detection rate of TTV; on the other hand, there was a positive correlation between the percentages of CD19 cells (B lymphocytes) and the detection rate of TTV [93]. Moreover, a role of TTV in the activity of eosinophils was suggested: in a group of children with ARD, the levels of serum eosinophil cationic protein (s-ECP) were significantly higher in children who were positive for the presence of TTV than those who were negative for the presence of TTV; in addition, an association was observed between TTV loads and the concentrations of s-ECP [37]. The authors suggested that TTV infection represented an “enhancer” or contributing factor which strengthened the release of ECP and other effector molecules. TTV infection could play a crucial role in airway hyperreactivity pathogenesis [37] given that in young children, high concentrations of s-ECP predict an increased likelihood of developing airway hyperreactivity and asthma later in life [93,94,95,96]. In patients with an allergic sensitization, TTV might worsen the extent and the severity of the allergic inflammatory response. More recently, an association was also found between TTV loads and exhaled nitric oxide, a sensitive marker of airway inflammation in asthmatic children [97].

Recent studies using molecular techniques have shown that antibiotics modified the composition of the pediatric cystic fibrosis (CF) microbiome, and, in addition, their repeated use also affected healthy airway commensals [98,99]. Willner et al. [99] assessed the changes in the distribution of viruses within two sets of CF lungs: in explanted lungs and in postmortem lungs; the virome in the right middle lobe of the postmortem lungs was mostly characterized by sequences closely related to TTV3.

Although the aforementioned studies found an association of TTV with chronic respiratory diseases and the impairment of lung function, the specific role of TTV in pediatric CF remains unknown [92].

Furthermore, Abbas et al. [76] showed that Anelloviridae was the first most frequent human DNA virus family revealed in 20 datasets among the most common human DNA virus families (Adenoviridae, Redondoviridae, Herpesviridae, Papillomaviridae, Parvoviridae, and Polyomaviridae), followed by Redondoviridae, a family of small, circular DNA viruses recently discovered [76]. Colonies of redondoviruses are found in human oro-respiratory sites and are involved in several human disorders, such as periodontitis and respiratory failure [76]. Noteworthy, only the presence of AVs was significantly concomitant with the presence of redondoviruses; the authors speculated that, probably, this concurrence of both viral families might be due to the inflammatory environment, which is similarly favorable for the replication of both, alternatively to a multiple displacement amplification which is enriched for both AVs and redondoviruses, leading to their better co-detection [76].

Finally, according to the aforementioned studies, it seems that TTV and other AVs can have a crucial role in lung function loss and asthma pathogenesis [55]. However, the evidence is not certain enough to establish that AVs can take a part in the development of chronic respiratory diseases, particularly in asthma. After this revision, what could we assume? We could certainly assume that an alteration in the normal lung virome occurs during asthma and other chronic respiratory diseases, but the pathogenetic role of AVs still remains unknown [55]. Further investigations are warranted to better define the spatial distribution and the composition of the virome in healthy subjects, the effects of the virome on the lungs, and the virome–host interactions. In conclusion, TTVs are associated with acute respiratory disease or exacerbated chronic lung disease [88,89], and lower CD3+ and CD4+ T cell numbers and higher B cell and eosinophilic counts [27,37] are associated with increased TTV loads. AVs probably have an immunomodulatory activity, and in a suppressed host immune system, they could promote the exacerbation of chronic pulmonary disease, as reported in COPD patients [89]. These findings could lay the groundwork to personalize immunosuppression and antimicrobial prophylaxis treatments. Additionally, a better comprehension of the all components of the virome could lead to the development of novel microbe-based therapies (i.e., probiotics) or biomarkers (TTV load) for the clinical management of CLDs in children, such as asthma.

## Figures and Tables

**Table 1:** Interaction between AVs and immune system.

**TTVs and Innate Immunity**
Interaction with PAMP receptors (PRR) and inflammasome
TTVs genome and CpGs stimulate TLR in infected cells: inflammatory cytokines (IFN-α, IL-6 and IL-12) are released
**TTVs and Adaptative Immunity**
ORF1 and ORF2 are recognized
TTV load inverse correlation with T lymphocytes
TTV load positive correlation with eosinophils

PAMP: Pathogen-associated molecular patterns; TTV: torque teno virus; PRR: pattern recognition receptors; CpGs: unmethylated heterodimers of guanosine and cytosine; TLR: Toll-like receptors; ORF: open reading frame.

**Table 2:** Potential escape mechanisms used by anelloviruses to elude the immune system.

Immuno-Escape Mechanisms
Inhibitory CpGs interaction with TLR
ORF2 inhibits NFkb translocation to the nucleus: IL-6, IL-8 and cyclooxygenase-2 are reduced
miRNA interaction with interferon antiviral pathway
Exosomes protect TTVs from neutralizing antibodies

TTV: torque teno virus; PRR: pattern recognition receptors; CpGs: unmethylated heterodimers of guanosine and cytosine; TLR: Toll-like receptors; ORF: open reading frame.
